# Effects of Neurophysiotherapy Based on Physical Activity on Cognitive and Psychosocial Functioning in Patients with Acquired Brain Injury

**DOI:** 10.3390/healthcare13202610

**Published:** 2025-10-16

**Authors:** Verónica Morales-Sánchez, Javier Cuesta-Aguilar, Daniel Asensio-Pérez, Desirée Gálvez-Guerrero, Lorena Morales-Blanca, Eva María Cubero-Lama, Gerardo Ricardo Moreu-Pérez-Artacho, Antonio Hernández-Mendo, Rafael E. Reigal

**Affiliations:** 1 Department of Social Psychology, Social Anthropology, Social Work and Social Services, University of Málaga, 29016 Málaga, Spain; 2 Association for Acquired Brain Injury of Málaga (ADACEMA), 29014 Málaga, Spain

**Keywords:** multidisciplinary intervention, cognitive recovery, emotional well-being, quality of life, neurorehabilitation

## Abstract

Introduction: Acquired brain injury (ABI) produces significant cognitive, motor, and psychosocial impairments that affect people’s daily functioning. Rehabilitation programs increasingly combine physical activity with neuropsychological strategies for greater effectiveness. Purpose: The aim of this study was to analyze the effects of neurophysiotherapy based on physical activity and neuropsychological rehabilitation on cognitive and psychosocial functioning in individuals with an acquired brain injury (ABI). Method: A total of 19 individuals between the ages of 24 and 89 years (*M* ± *SD*: age = 59.26 ± 19.01) belonging to the Acquired Brain Injury Association of Málaga (ADACEMA) participated in this study. A quasi-experimental design with pre- and post-test measures and multiple experimental groups was used. The instruments used were the digit subtest of the Barcelona Test, the Five Digit Test (FDT), the Tower of Hanoi, the modified six-element subtest of the Behavioural Assessment of the Dysexecutive Syndrome, the Trail Making Test (TMT), the WHOQOL-BREF, and the Profile of Mood States (POMS) questionnaire. The Kruskal–Wallis H, Mann–Whitney U, and Wilcoxon tests were used to analyze the data. Results: The results obtained showed a positive effect of physical activity (PA) combined with neuropsychological rehabilitation on working memory, planning, emotional well-being, personal relationships, depressive symptoms, and overall quality of life. Conclusions: The findings suggest that combining neurophysiotherapeutic physical-activity-based rehabilitation with other neuropsychological interventions may be a promising approach to improving executive functioning, emotional well-being, and quality of life in people with an ABI. These preliminary results highlight the potential value of multidisciplinary programs in post-injury recovery, although further studies with larger and more homogeneous samples are needed to confirm these effects.

## 1. Introduction

Brain damage refers to an alteration in brain function that leads to neurological dysfunction, with consequences for cognition, mood, and daily life functioning [1,2,3,4]. Due to the complexity of its etiology and clinical course, people with brain damage require comprehensive care addressing multiple needs [5]. Specifically, acquired brain injury (ABI) is used to refer to injuries that appear after birth and are not caused by hereditary or congenital diseases [6]. The most prevalent injuries produced by ABI are traumatic, such as traumatic brain injury, which has the highest incidence worldwide, with more than 259 cases per 100,000 inhabitants per year [7], but can also be atraumatic, such as stroke, which has the second highest incidence worldwide, with up to 141 cases per 100,000 inhabitants per year [8].

On the other hand, due to the importance of the brain in control and functioning of the organism, the consequences of an ABI can be diverse [9], affecting neuro-cognitive, motor, psychoemotional, and behavioral processes [10]. In relation to neuro-cognitive processes, disorders of attention, memory (amnesia, short- and long-term, working memory), and language (aphasia) and executive function impairments are observed [11]. Regarding psychoemotional symptoms, anxiety, depression, low self-perception and/or self-efficacy, and a decreased perception of quality of life may be present [12]. Regarding motor function, apraxia, hemiplegia, hemiparesis, spasticity, and fatigue are among the most common effects, e.g., [13,14,15].

This impact is clearly reflected in activities of daily living [16], with studies reporting a decrease in functional efficiency among people with ABIs [17], as well as in intellectual, cognitive, and behavioral performance [6]. Consequently, research in recent years has increasingly focused on rehabilitation programs aimed at improving daily functioning [18].

While it is true that the implementation of a rehabilitation program is essential for patients with ABI, defining it is a complex process due to the heterogeneity and breadth of both lesions and the alternatives in programs. However, there seems to be a consensus on the multidisciplinary nature they should acquire, adopting a comprehensive and multifaceted approach where the patient, the family environment, and the therapeutic team are included [19,20]. Today, it is still a complicated task to prescribe adequate and effective rehabilitation treatments for people with an ABI [21]. Specifically, there remains a gap in how to effectively integrate and evaluate combined neurophysiotherapeutic and neuropsychological interventions and their specific impact on executive functioning, emotional well-being, and daily functioning in people with ABIs.

Recent findings have shown that psychological treatments are effective in improving some psychoemotional aspects [22]. In turn, psychosocial function, which seems to be guided in these cases by environmental accessibility and patient motivation, is an effective element in post-injury recovery [23]. Similarly, neuropsychological rehabilitation has traditionally been the cornerstone of these processes, improving psychological well-being, anxiety, depression, and quality of life [24].

For its part, the recent literature has highlighted that the implementation of physical activity (PA) programs in physiotherapeutic and neuro-cognitive rehabilitation is increasingly recognized as a valuable strategy for the improvement of functional, cognitive, and psychosocial recovery processes in patients with ABIs [14]. Several studies have observed positive changes in many of the concurrent effects of an ABI after the implementation of a PA program [25], although more evidence is still needed to define the typology of the exercises, as well as the volume and intensity of these exercises [26].

Thus, this issue is of great therapeutic and social importance given the impact of ABI on the daily functioning of people who suffer from it. Furthermore, it has been observed that rehabilitation is increasingly aimed at generating integrated programs enabling multidisciplinary action to address the functional deficits generated by ABI. Hence, the present study attempts to address how PA in neurophysiotherapy can be an element that contributes to the recovery processes of people with ABIs. In this sense, PA could improve the movement patterns of these patients; in parallel, cognitive functioning; and, in general, their psychosocial well-being and quality of life. Therefore, the aim of this study was to analyze the effects of neurophysiotherapeutic rehabilitation combining physical activity and a neuropsychological intervention on cognitive and psychosocial functioning in patients with an acquired brain injury. Thus, the research hypothesis was that statistically significant improvements in the cognitive and psychosocial functioning of patients with an acquired brain injury would be determined when physical activity was included in the rehabilitation program, compared to neuropsychological rehabilitation alone. There is still limited evidence on the effects of combining physical activity with neuropsychological rehabilitation. This study contributes to the growing but scarce literature exploring such integrated interventions in patients with ABIs.

## 2. Materials and Methods

### 2.1. Design

This research followed a manipulative, quasi-experimental strategy with several intervention groups [27].

### 2.2. Participants

The study included 12 men and 7 women aged between 24 and 89 years (*M* ± *SD*: age = 59.26 ± 19.01). The sample was selected from the Acquired Brain Injury Association of Malaga (ADACEMA, Malaga, Spain). All participants were in the stabilization phase of their injury, which had occurred at least 12 months prior. We included users who had suffered some type of ABI and who attended neurophysiotherapy and/or neuropsychology sessions at least once a week. Users whose functional and/or cognitive status prevented them from completing the protocols or tests, or who did not have continuity in the intervention programs, were not included. The division into each group was made taking into account the Modified Rankin Scale (mRS), a scale that measures the degree of dependence of the subject [28], with the intention of generating groups with the greatest homogeneity among them. In addition, the Glasgow Coma Scale (GCS) was employed to categorize the severity of traumatic brain injury (TBI) among participants. Table 1 summarizes the medical diagnoses and baseline characteristics of the participants included in the study.

### 2.3. Instruments

#### 2.3.1. Executive Function

1. Direct and inverse digit subtest of the Barcelona Test [29]: This test allows for a highly sensitive assessment of working memory and has been widely used in the field of neuropsychology to detect brain pathologies [30]. It consists of repeating orally a series of digits that increase in quantity, first directly and then inversely.
2. Five-digit test [31]: This test was used to assess inhibitory control and consists of four tests: reading and counting, referring to automatic processes, and choice and alternation, referring to controlled processes. The last two tests are the most rigorous in assessing the abilities evaluated by the test.
3. The Tower of Hanoi [32]: This puzzle has been widely used in the field of neuropsychology to assess executive functioning in traumatic injuries, specifically planning ability [33]. Moreover, it represents a test of high accessibility and easy administration.
4. The modified six-item subtest of the Behavioural Assessment of Dysexecutive Syndrome (BADS) [34,35]: This test detects prefrontal syndrome, with the prefrontal area of the brain involved in dual execution, planning, organization, and problem solving. It consists of working autonomously for 10 min completing three types of tasks (dictation, arithmetic problems, and image naming), which are subdivided into part A and part B, with a series of rules whose violation influences the test score. The multiplicity of processes involved in this test makes it particularly sensitive to executive functions.
5. Trail Making Test (tests A and B) [36]: This test, which evaluates cognitive flexibility and processing speed, consists of tracing trails through a set of numbers for part A (from 1 to 25) and a series of numbers–letters for part B (from 1-A to 12-L) with an established pattern. It has been widely used in the field of neuropsychology since its appearance [37] and also with a population similar to that of the present study [38,39].

#### 2.3.2. Psychosocial Aspect Evaluation

1. The CAVIDACE test [40]: Specifically, the Emotional Well-being and Personal Development subscales were used. It is an instrument designed and validated specifically for this type of population that is used to measure quality of life in people with ABI from a comprehensive perspective, taking into account the context and personal factors of each individual [41]. The internal consistency values (Cronbach’s Alpha) were between 0.78 and 0.85.
2. The quality of life test proposed by the World Health Organization, WHOQOL-BREF [42], as the abbreviated version adapted to Spanish [43]. This test provides a profile of the subjective perception of global quality of life and general health. In addition, it has several subscales that refer to physical health, psychological aspects, personal relationships, and environment. The internal consistency values (Cronbach’s Alpha) were between 0.81 and 0.92.
3. The brief version adapted to Spanish of the Profile of Mood States (POMS) questionnaire [44]: This questionnaire was used to assess mood. It consists of assessing with a 4-point Likert-type scale 30 items referring to 6 scales (Anger, Fatigue, Vigor, Friendliness, Tension, and Depression). The internal consistency values (Cronbach’s Alpha) were between 0.76 and 0.89.

### 2.4. Procedure

A collaboration agreement was established with ADACEMA, and the pertinent permissions were requested, as well as the informed consent of each participant. During this study, the ethical principles of the Declaration of Helsinki were respected, and the research was approved by the Experimentation Ethics Committee of the University of Malaga (CEUMA-33-2023-H).

Three intervention groups were initially generated according to the rehabilitation protocol to be followed: (A), which performed neurophysiotherapy sessions based on physical activity and neuropsychology; (B), which performed neurophysiotherapy sessions based on physical activity; and (C), which performed neuropsychology sessions. In addition, the physical-activity-based neurophysiotherapy sessions in groups A and B were subdivided further into two modalities: groups A1 and B1 (Conventional Neurophysiotherapy—CN) and groups A2 and B2 (Conventional Neurophysiotherapy and Repetitive Tasks—CN+RT). This subdivision generated the possibility of comparison between five groups. Two psychosocial and neuropsychological assessments were conducted, one at the baseline and another at the end of the 12-week program.

#### 2.4.1. Conventional Neurophysiotherapy Protocol (CN) (Groups A1 and B1)

It consisted of five PA-based therapeutic blocks with variable amounts of time spent depending on the patient. However, the average times were gait training (26%), strength work (21%), manual therapy (15%), balance and coordination work (24%), and transfer training (14%). Two weekly sessions per patient of 50 min were performed per week aiming to improve the quality of life of users following the recommendations of the Practical Guide for Stroke Rehabilitation by Winstein et al. [45] and the Galician Society of Internal Medicine [46].

#### 2.4.2. Conventional Neurophysiotherapy and Repetitive Task Training Protocol (CN+RT) (Groups A2 and B2)

This consisted of repetitive and intensive work on an individualized exercise aiming to improve a specific phase of gait that the participant had not yet mastered, following the recommendations and criteria described by French et al. [47]. It consisted of two phases that the patient overcame as he or she correctly executed the movement and incorporated it into his or her daily gait. There was a high degree of individualization, which is reflected in Appendix A (see at the end of the document). There were three 50 min sessions per patient per week, of which 30 min was devoted to NFC and the remaining 20 to TR.

#### 2.4.3. Neuropsychology Protocol (Groups A and C)

This consisted of tasks focused on the improvement of executive functions through processing speed exercises, working memory, lexical access, abstraction or cognitive flexibility, and, on the other hand, psychological therapies. To minimize fatigue, the intervention was delivered in two individual 50 min sessions per week. All tasks were administered in a quiet room with adequate lighting and free from distractions. Before each task, the researcher carefully explained the instructions to ensure full understanding of the protocol, and brief pauses were incorporated when necessary to maintain the optimal engagement and performance.

#### 2.4.4. Data Analysis

Descriptive and inferential analyses were performed. Means, standard deviations, skewness, and kurtosis were analyzed. In addition, the Shapiro–Wilk test was used to evaluate the normality of the data distribution. The Kruskal–Wallis H, Mann–Whitney U, and Wilcoxon nonparametric tests were used to evaluate the efficacy of the interventions performed. Effect sizes were calculated to estimate the magnitude of the differences observed using the corresponding formulas. For the Kruskal–Wallis test, eta squared (*η*^2^*_h_* = (*H* − *k* + *1*)/(*n* − *k*)) was used, whereas for the Mann–Whitney U and Wilcoxon tests, effect sizes (*r*) were computed as *r* = *Z*/*√N*. According to Cohen’s guidelines, values of 0.1, 0.3, and 0.5 correspond to small, medium, and large effects, respectively. However, due to the small sample size and the exploratory nature of this study, these estimates should be interpreted with caution and considered indicative rather than conclusive. The level of significance was set at α = 0.05. IBM SPSS v. 23 software was used for data processing.

## 3. Results

### 3.1. Analysis of the Results According to Three Groups

First, the differences between groups A (neurophysiotherapy and neuropsychology), B (neurophysiotherapy), and C (neuropsychology) were analyzed, without taking into account the division of the interventions of groups A and B into CN and CN+RT. Table 2 and Table 3 show the means and standard deviations of the study variables for the three groups. In addition, the skewness values ranged from −2.23 to 2.04, and the kurtosis values ranged from −4.89 to 5.01. Given the values of the Shapiro–Wilk test, which were statistically significant in most cases, and the sample size in each group, we opted to perform nonparametric tests to analyze the differences between and within groups.

The Kruskal–Wallis and Mann–Whitney U tests were used to evaluate the differences between groups pre and post assessments. There were only differences between groups pre assessment for the variables physical health (*𝒳*^2^ = 11.35, *p* < 0.05) and psychological aspects (*𝒳*^2^ = 10.70, *p* < *0*.05) of the WHOQOL-BREF, as well as in Vigor (*𝒳*^2^ = 10.05, *p* < *0*.05) as assessed using the POMS. On the other hand, post assessment, there were differences in the variables physical health (*𝒳*^2^ = 9.81, *p* < *0*.05) from the WHOQOL-BREF and in Vigor (*𝒳*^2^ = 10.43, *p* < *0*.05) from the POMS. Specifically, differences were established in all cases between groups A and C at a *p* < *0*.05 level. The effect size estimates for the Kruskal–Wallis tests (η^2^_h_ ≈ 0.45–0.50) indicated large magnitudes of group differences.

In turn, Wilcoxon’s test was used to analyze the pre and post differences in each of the groups. There were no differences in the group that performed only neuropsychological rehabilitation (group C). There were only differences in the Tower of Hanoi (seconds) (*Z =* −2.20, *p* < *0*.05) in the group performing only physical-activity-based neurophysiotherapy (group B). Statistically significant differences were found in the group receiving neurophysiotherapy based on physical activity combined with neuropsychological intervention (group A) in more tests, specifically in the inverse digit subtest of the Barcelona Test (*Z =* −2.23, *p* < 0.05), the Tower of Hanoi (seconds) (*Z =* −2.36, *p* < 0.01), emotional well-being (CAVIDACE) (*Z =* −2.44, *p* < 0.01), BADS (*Z =* −2.06, *p* < 0.05), personal relationships (the WHOQOL) (*Z =* −2.20, *p* < *0*.05) and depression (POMS) (*Z =* −2.03, *p* < 0.05). The corresponding effect sizes for Wilcoxon’s tests (r range = 0.68–0.79) reflected large effects.

### 3.2. Analysis of the Results According to Five Groups

After analyzing the differences between groups A, B, and C, we wanted to evaluate whether the subdivisions of the neurophysiotherapy interventions (CN and CN+RT) generated statistically significant differences. Therefore, the differences between five groups were analyzed: A1 = CN and neuropsychological sessions; A2 = CN + RT and neuropsychological sessions; B1 = CN sessions; B2 = CN + RT; and C = neuropsychological sessions.

Table 4 and Table 5 show the means and standard deviations of the study variables. In addition, the values of skewness ranged, as a whole, between −0.70 and 3.98, and those for kurtosis ranged between −1.54 and 15.92. In addition, the values of the Shapiro–Wilk test were significant in most cases. For this reason, and given the sample size, we chose to perform nonparametric tests for inter- and intra-group comparisons.

The Kruskal–Wallis and Mann–Whitney U tests performed indicated that there were only pre-test differences between groups on the physical health (*𝒳*^2^ = 11.35, *p*< 0.05) and psychological aspects (*𝒳*^2^ = 10.69, *p* < 0.05) subscales (WHOQOL), as well as for Vigor (*𝒳*^2^ = 10.05, *p* < 0.05) (POMS). Specifically, for physical health, differences were found between groups A1 and B1; A2 and C; B1 and B2; and B1 and C (*p* < 0.05). For psychological aspects, differences were observed between groups A1 and B2; B1 and B2; and B1 and C (*p* < 0.05). For the Vigor scale, differences were obtained between groups A1 and B2; A2 and B2; A2 and C; and B1 and B2 (*p* < 0.05). The effect sizes for the Kruskal–Wallis tests in this analysis (η^2^_h_ ≈ 0.42–0.47) also indicated large effects.

Likewise, there were only post-test differences in physical health (*𝒳*^2^ = 9.82, *p* < 0.05) (WHOQOL) between groups A1 and B1; B1 vs. B2; and B1 and C (*p* < 0.05) and in vigor (*𝒳*^2^ = 10.43, *p* < 0.05) (POMS) between groups A2 and B1; B1 and B2; and B1 and C (*p* < *0*.05). Post hoc Mann–Whitney U comparisons yielded effect sizes in the large range (r = 0.52–0.65), confirming the robustness of the between-group differences.

On the other hand, the results of Wilcoxon’s test indicated that there were only differences between pre and post evaluation in group A1 in the emotional well-being subscale of the CAVIDACE test (*Z =* −2.02, *p* < 0.05). The corresponding effect size (r = 0.67) indicated a large effect, suggesting a meaningful improvement in emotional well-being.

## 4. Discussion

The aim of this research was to evaluate the effects of neurophysiotherapy rehabilitation based on physical activity (PA) and neuropsychology on cognitive and psychosocial functioning in patients with an acquired brain injury (ABI). For this purpose, the differential impact of a combined program of neurophysiotherapy with PA and neuropsychology was analyzed, compared to other programs in which only neurophysiotherapy with PA or neuropsychology was performed. In general terms, the findings have shown that the combination of neurophysiotherapy with PA together with neuropsychology has been the most effective procedure for cognitive and psychosocial functioning in the study sample. However, these results should be interpreted with caution, as several limitations, such as sample size and heterogeneity, prevent firm conclusions from being drawn. Therefore, due to these issues, which may generate interpretation biases, the analyses should be considered exploratory and the findings preliminary.

First, analyses were performed among three groups to evaluate whether the combined intervention (neurophysiotherapy + neuropsychology) generated statistically significant effects in patients with an ABI. The results showed significant changes in the parameters of working memory (inverse digits), execution speed, emotional well-being, planning, personal relationships, and depression and were close to being significant in working memory (direct digits), cognitive flexibility, physical health, tension, and anger in the combined groups. This is consistent with previous studies that have highlighted the importance of promoting these types of multidisciplinary interventions for achieving more significant effects [48,49]. In fact, previous research had highlighted the effects of physical and motor interventions on several variables of cognitive and psychological functioning [24,25,26,50]. Thus, the results suggest that the inclusion of programs where physical activity is performed, together with other therapies, could be an excellent option for improving the situations of these patients.

The present study conducted interventions with patients with chronic injuries, highlighting the importance of these types of programs. However, these findings suggest that these programs could also be implemented at other stages of the injury, including those which may be more conducive to improving recovery outcomes. In fact, there are positions that point out the importance of intervention from the beginning of the injury—that is, starting in the acute phase of the injury and in the hospital context, due to the positive effects that movement could have in a phase in which inactivity and social isolation could be a detrimental element in the phase of spontaneous recovery from the injury [51]. In addition, it could also be beneficial due to the environmental enrichment that is achieved, avoiding boredom and its negative effects [52], as well starting to introduce the patient into a social environment, which will bring benefits at a psychoemotional level [53]. Studies like this one highlight that even in the stabilization phase, these types of interventions can help improve patients’ expectations for improvement, opening up the possibility of studying how these individuals can benefit from these combined therapies to improve their health and functional capacity.

Secondly, to determine the differences between two types of neurophysiotherapy programs, five group analyses were performed. These consisted of one focused entirely on neurophysiotherapeutic rehabilitation and another focusing a portion of the session on repetitive gait task training. When analyses were carried out in five groups, the data revealed that repetitive task training does not seem to be a determining type of exercise for cognitive and psychosocial improvements in people suffering from stroke, traumatic brain injury, or brain tumors. The results did not confirm that dedicating part of the rehabilitation time to this type of exercise could provide greater benefits in terms of the patients’ cognitive and psychosocial functioning. On the other hand, the results have corroborated that as long as there is physical activity in neurophysiotherapy sessions, the improvements will be more significant and seen for a wider range of variables than in the implementation of programs consisting solely of neuropsychological rehabilitation.

These results may have been influenced by some of the limitations of this study. First, the small sample size and the lack of random group assignment could lead to statistical and interpretation errors due to measurement bias. For example, the reduced number of participants could have limited the statistical effects of the program. Furthermore, subgrouping should be complemented by adjustments for multiple comparisons, reducing the potential inclusion of false positives in the analyses. However, this is a very specific sample, which makes it difficult to recruit participants. Second, another limitation has been the heterogeneity of the sample. ABI is an umbrella term that encompasses a wide variety of lesions, which makes it difficult to draw solid conclusions about the results obtained. Moreover, not all detailed diagnostic information (e.g., laterality or lobar involvement) was consistently available in the medical records of all participants, which may restrict comparability across cases, although the data collected still provide valuable insights into rehabilitation outcomes. In future studies, we suggest increasing the sample size and standardizing the sample either according to the type of ABI or the patients’ injuries, as well as verifying whether another type of physical activity could be more beneficial for post-injury recovery. Specifically, an important limitation of the present study is the wide age range of participants, which included both younger and older adults with acquired brain injuries. Although elderly patients could be considered a distinct subgroup due to age-related cognitive differences, the limited sample size did not allow us to perform separate analyses. Future studies with larger samples should stratify participants by age to examine potential differences between younger and geriatric populations in their neurorehabilitation outcomes. Finally, there was no follow-up evaluation, which limits knowledge about the long-term impact of program implementation. Future research should include larger, randomized samples and follow-up assessments to confirm and extend the present findings.

## 5. Conclusions

Acquired brain injury is a broad and heterogeneous reality that makes scientific progress towards solid conclusions difficult. However, in line with other studies, the present research has highlighted the importance of movement and physical activity in neurophysiotherapy for this pathology, showing improvements across a wide variety of executive and psychoemotional functions in people with an acquired brain injury. Neurophysiotherapy based on physical activity is a promising strategy for improving outcomes in people with an acquired brain injury.

## Figures and Tables

**Table 1:** Medical diagnoses and baseline characteristics of the participants.

ID	Age (Years)	Sex	Diagnosis	mRS (0–6)	GCS	Education Level	Intervention Group
1	24	M	HS	4	14	S	A1
2	56	F	IS	3	15	S	A1
3	74	M	HS	4	14	U	A1
4	77	M	TBI	2	14	U	A1
5	85	M	IS	4	14	S	A1
6	58	F	IS	4	15	U	A2
7	67	M	HS	2	15	S	A2
8	45	F	HS	4	13	U	A2
9	69	F	IS	2	13	U	A2
10	66	F	HS	3	15	U	B1
11	70	M	BT	4	13	S	B1
12	45	M	HS	3	15	U	B1
13	87	F	IS	3	15	U	B2
14	47	M	TBI	4	14	S	B2
15	35	M	TBI	4	13	S	B2
16	89	F	IS	3	11	P	B2
17	30	M	BT	2	15	U	C
18	53	M	HS	2	15	U	C
19	49	M	TBI	3	14	S	C

Note: M = male; F = female; HS = hemorrhagic stroke; IS = ischemic stroke; TBI = traumatic brain injury; BT = brain tumor; U = university; S = secondary; P = primary; mRS = Modified Rankin Scale; GCS = Glasgow Coma Scale.

**Table 2:** Means and standard deviations for executive functioning (3 groups).

	Group A (*n* = 9)		Group B (*n* = 7)		Group C (*n* = 3)	
	M	SD	M	SD	M	SD
BT-Direct Digits-Pre	5.20	0.84	5.75	0.95	6.00	1.73
BT-Digits Direct-Post	5.80	0.44	5.75	0.95	5.67	1.53
BT-Inverse Digits Pre	3.20	0.83	3.75	0.95	4.67	1.52
BT-Inverse Digits Post	4.40	1.51	4.00	0.81	4.67	1.52
TMT-A_pre	132.00	57.90	73.00	31.18	76.00	63.32
TMT-A_post	117.60	91.63	76.25	36.13	79.33	61.33
TMT-B_pre	327.40	172.99	299.00	222.42	118.00	62.00
TMT-B_post	308.60	180.90	288.25	214.29	137.00	82.82
T. Hanoi (sec) pre	193.40	231.30	207.50	261.91	81.33	42.36
T. Hanoi (sec) post	46.60	22.14	76.75	49.01	120.33	104.83
T. Hanoi (mov) pre	14.60	5.72	16.75	8.18	12.00	6.24
T. Hanoi (mov) post	10.00	4.12	13.25	6.13	13.67	11.54
FDT-Inhibition-pre	6.20	2.86	7.25	2.50	6.67	2.08
FDT-Inhibition-post	7.00	2.91	8.50	0.70	7.00	3.00
FDT-Flexibility-pre	5.80	2.58	7.25	2.75	5.67	2.88
FDT-Flexibility-post	7.40	2.60	5.50	2.38	7.67	2.31

Note: M = mean; SD = standard deviation; BT = Barcelona Test; TMT = Trail Making Test; T. Hanoi = Tower of Hanoi; FDT = five-digit test.

**Table 3:** Means and standard deviations for the psychosocial domain (3 groups).

	Group A (*n* = 9)		Group B (*n* = 7)		Group C (*n* = 3)	
	M	SD	M	SD	M	SD
CAVIDACE-EW-pre	16.71	4.07	17.80	3.70	15.67	6.42
CAVIDACE-EW-post	19.29	2.36	20.40	1.82	18.67	1.52
CAVIDACE-PD-pre	16.43	2.76	13.60	3.21	16.67	2.88
CAVIDACE-PD-post	17.29	2.75	16.40	3.91	16.67	7.10
BADS-pre	1.86	1.57	2.00	2.34	1.00	1.00
BADS-post	3.43	2.22	2.60	2.30	0.67	0.57
WHOQOL-Total-pre	87.43	11.01	96.80	15.78	99.33	6.64
WHOQOL-Total-post	93.71	7.50	98.60	15.66	102.0	14.00
WHOQOL-PH-pre	20.29	3.45	22.60	5.56	28.33	2.08
WHOQOL-PH-post	25.43	2.37	24.20	3.83	28.33	3.78
WHOQOL-Psi-pre	21.14	2.96	23.80	3.90	24.00	1.00
WHOQOL-Psi-post	22.14	2.80	23.00	4.30	23.00	4.58
WHOQOL-Rel-pre	9.14	2.41	11.00	2.56	10.67	1.52
WHOQOL-Rel-post	11.14	1.86	11.00	1.87	10.33	1.53
WHOQOL-Env-pre	28.14	4.33	31.80	5.31	32.33	2.51
WHOQOL-Env-post	28.29	2.43	32.40	4.33	33.00	4.36
POMS-Tension-pre	4.43	4.90	5.40	4.51	0.67	0.57
POMS-Tension-post	2.71	3.40	3.40	4.10	2.67	3.06
POMS-Depression-pre	5.29	6.21	2.20	2.96	1.33	1.53
POMS-Depression-post	3.00	2.94	1.80	1.64	2.33	3.21
POMS-Cholera-pre	3.14	5.00	3.60	4.50	3.90	4.50
POMS-Cholera-post	2.00	3.31	2.00	1.87	1.33	1.53
POMS-Vigor-pre	10.14	3.67	14.40	2.88	14.67	3.78
POMS-Vigor-post	13.00	2.31	14.00	4.00	13.67	2.08
POMS-Fatigue-pre	7.14	4.71	6.00	5.87	9.80	1.78
POMS-Fatigue-post	5.71	3.81	3.80	3.70	1.67	2.88
POMS- Friendliness -pre	14.43	3.50	16.40	2.60	15.33	0.57
POMS-Friendliness-post	14.00	2.08	15.40	3.36	13.67	3.21

Note: M = mean; SD = standard deviation; EW = emotional well-being; PD = personal development; PH = physical health; Psi = psychological aspects; Rel = personal relationships; Env= environment.

**Table 4:** Means and standard deviations for executive functioning.

	Group A1 (*n* = 5)		Group A2 (*n* = 4)		Group B1 (*n* = 3)		Group B2 (*n* = 4)		Group C (*n* = 3)	
	M	SD	M	SD	M	SD	M	SD	M	SD
TB-Direct Digits-Pre	5.40	0.89	4.00	0.81	6.67	0.57	4.75	1.26	6.00	1.73
TB-Digits Direct-Post	5.80	1.09	5.00	1.41	6.00	1.00	5.00	0.82	5.67	1.52
TB-Inverse Digits Pre	2.80	0.83	3.25	0.95	4.00	1.00	3.00	0.81	4.67	1.52
TB-Inverse Digits Post	4.00	0.70	4.25	2.06	4.00	1.00	3.25	0.95	4.67	1.52
TMT-A_pre	228.00	159.65	115.33	75.72	120.67	139.75	83.00	29.30	76.00	63.31
TMT-A_post	222.50	153.26	71.33	36.47	90.67	74.00	84.33	39.57	79.33	61.33
TMT-B_pre	390.33	211.52	221.00	26.23	82.00	65.81	371.33	206.92	118.00	62.00
TMT-B_post	383.33	210.31	184.33	26.76	114.00	32.47	346.33	220.54	137.00	82.81
T.Hanoi (sec) pre	337.25	305.97	112.33	27.61	345.50	359.91	334.75	306.33	81.33	42.36
T.Hanoi (sec) post	107.00	143.29	67.33	8.73	111.50	68.58	85.25	48.78	120.33	104.83
T.Hanoi (mov) pre	11.75	4.19	16.33	5.85	313.50	405.17	10.50	7.23	12.00	6.24
T.Hanoi (mov) post	12.50	5.26	8.00	1.73	18.50	3.53	13.25	6.13	13.67	11.54
FDT-Inhibition-pre	5.25	3.59	6.50	0.70	5.00	4.24	7.00	3.00	0.00	0.01
FDT-Inhibition-post	4.25	2.98	9.50	0.70	5.00	4.24	8.00	0.00	1.67	2.88
FDT-Flexibility-pre	5.25	3.30	5.50	0.70	6.00	4.24	6.67	3.05	15.67	6.42
FDT-Flexibility-post	5.50	4.20	8.00	0.01	6.50	3.53	4.33	0.57	18.67	1.52

Note: M = mean; SD = standard deviation; TB = Barcelona Test; TMT = Trail Making Test; FDT = five-digit test.

**Table 5:** Means and standard deviations for psychosocial domains.

	Group 1 (*n* = 5)		Group 2 (*n* = 4)		Group 3 (*n* = 3)		Group 4 (*n* = 4)		Group 5 (*n* = 3)	
	M	SD	M	SD	M	SD	M	SD	M	SD
CAVIDACE-EW-pre	14.60	1.51	18.50	4.50	15.67	4.61	18.75	2.75	15.67	6.42
CAVIDACE-EW-post	17.60	1.34	20.50	2.38	17.00	3.60	21.25	1.25	18.67	1.52
CAVIDACE-PD-pre	15.40	3.84	17.00	3.36	14.00	1.00	12.75	4.11	16.67	2.88
CAVIDACE-PD-post	16.20	1.92	18.50	3.00	14.67	4.16	17.25	3.09	16.67	7.09
BADS-pre	1.67	2.08	2.00	1.41	2.50	3.53	1.67	2.08	1.00	1.00
BADS-post	2.33	3.21	4.25	0.95	3.50	3.53	2.00	1.73	0.67	0.57
WHOQOL-Total-pre	86.20	12.65	88.00	6.97	74.33	15.04	102.50	10.24	99.33	6.65
WHOQOL-Total-post	89.40	7.70	96.00	5.88	76.67	14.29	103.25	13.52	102.00	14.00
WHOQOL-PH-pre	22.80	3.89	19.50	4.20	17.33	2.30	25.50	3.87	28.33	2.08
WHOQOL-PH-post	24.00	3.67	25.50	.57	18.67	3.21	26.00	2.44	28.33	3.78
WHOQOL-Psi-pre	20.00	2.91	22.00	2.16	17.33	4.61	25.25	2.98	24.00	1.00
WHOQOL-Psi-post	20.40	2.30	23.00	2.94	17.00	3.60	24.75	3.09	23.00	4.58
WHOQOL-Rel-pre	8.00	1.41	10.00	2.58	7.67	2.30	12.25	2.06	10.67	1.52
WHOQOL-Rel-post	10.20	1.48	12.25	1.25	8.00	2.64	11.50	1.73	10.33	1.52
WHOQOL-Env-pre	27.20	4.43	30.25	4.78	28.00	5.56	32.00	5.41	32.33	2.51
WHOQOL-Env-post	28.00	1.58	29.00	2.94	27.00	4.00	32.75	4.92	33.00	4.35
POMS-Tension-pre	3.40	2.19	4.50	6.60	10.33	.57	2.33	2.30	0.67	0.57
POMS-Tension-post	1.80	1.09	3.00	4.76	8.00	3.46	1.00	1.73	2.67	3.05
POMS-Depression-pre	5.80	3.56	5.25	8.61	8.67	6.65	0.33	0.57	1.33	1.52
POMS-Depression-post	3.00	1.58	2.25	3.86	7.00	7.93	1.33	1.52	2.33	3.21
POMS-Cholera-pre	2.40	2.51	4.00	6.16	10.00	5.56	1.00	1.73	0.00	0.00
POMS-Cholera-post	1.20	1.30	2.25	4.50	6.33	4.93	1.00	1.73	1.33	1.52
POMS-Vigor-pre	10.00	4.69	8.25	3.86	8.00	6.08	16.33	1.52	14.67	3.78
POMS-Vigor-post	10.60	3.91	13.50	1.73	7.67	4.16	16.67	2.08	13.67	2.08
POMS-Fatigue-pre	6.80	3.90	8.00	5.41	9.67	2.51	4.33	7.50	0.00	0.00
POMS-Fatigue-post	4.40	2.51	6.00	4.76	8.67	2.51	1.33	1.52	1.67	2.88
POMS- Friendliness -pre	14.80	0.447	14.25	4.92	12.33	4.04	17.67	2.30	15.33	0.577
POMS-Friendliness-post	13.40	2.30	14.50	1.00	11.33	3.51	17.00	3.00	13.67	3.21

Note: M = mean; SD = standard deviation; EW = emotional well-being; PD = personal development; PH = physical health; Psi = psychological aspects; Rel = personal relationships; Env = environment.

## Data Availability

The data are available upon request to the authors.

## Appendix A

Individualized phases of the NFC+TR program.

	**OBJ PHASE 1**	**OBJ PHASE 2**
GROUP A2		
6	Affected heel support	Support foot affection
7	Step foot affection	Passage length
8	Support foot affection	X
9	Step foot affection	Weight transfer
GROUP B2		
13	Support foot affection	X
14	Weight transfer	Support foot affection
15	I pass with affection	X
16	I pass with affection	X
Note: X = The objective of phase 1 was not achieved, and work continued based on the same objective in phase 2.		
